# Photoacoustic Tomography Tracks Vascular Remodeling and Functional Recovery in Liver Regeneration

**DOI:** 10.34133/bmef.0327

**Published:** 2026-10-21

**Authors:** Fan Meng, Xiali Gao, Hao Huang, Zhibo Xiao, Xuran Zhou, Yiyin Su, Ruimin Chen, Yang Xiao, Dikui Zhou, Yuqi Wang, Xiangdi Li, Xuanhao Wang, Junhui Shi

**Affiliations:** ^1^ College of Biomedical Engineering and Instrument Science, Zhejiang University, Hangzhou 310027, China.; ^2^ Zhejiang Lab, Hangzhou 311121, China.; ^3^ State Key Laboratory of Ocean Sensing, Zhejiang University, Hangzhou 310058, China.

## Abstract

**Objective:** This study aims to longitudinally monitor hepatic vascular remodeling and functional recovery during liver regeneration in mice using 3-dimensional panoramic photoacoustic computed tomography (3D-PanoPACT). **Impact Statement:** By enabling noninvasive, whole-organ spatiotemporal tracking within the same animal, this work establishes a novel imaging framework for staging organ regeneration and mapping vascular dynamics. **Introduction:** Hepatic vascular remodeling is critical for successful liver regeneration; however, existing imaging modalities lack the capacity for noninvasive, longitudinal, 3D characterization at the whole-organ scale. **Methods:** Male nude mice under isoflurane anesthesia underwent 70% partial hepatectomy or served as controls. A custom 3D-PanoPACT system performed continuous whole-organ imaging over 16 d. Quantitative vascular parameters, contrast clearance kinetics, and histological validation via hematoxylin and eosin and CD31 staining were assessed. Serum biochemical analyses independently evaluated hepatobiliary functional recovery. **Results:** 3D-PanoPACT delineated 3 temporally distinct vascular remodeling phases: proliferative (days 2 to 4), recovery (days 5 to 9), and remodeling (days 10 to 16). By day 16, regenerated livers exhibited no significant differences from normal controls in vascular volume, vessel length, mean diameter, or branching density, which was further confirmed by CD31 evaluation. While functional imaging showed a slightly delayed early peak enhancement, long-term metabolic clearance remained similar to that of normal controls. This functional recovery was independently corroborated by normal-like serum biochemical profiles at day 16. **Conclusion:** 3D-PanoPACT enables noninvasive, longitudinal tracking of hepatic regeneration. By delineating distinct morphological phases and complementing them with metabolic and biochemical validations, this framework effectively maps macroscopic vascular remodeling and confirms adequate functional recovery by day 16.

## Introduction

The liver is the largest metabolic organ in vertebrates, performing essential functions in detoxification, protein synthesis, and metabolic homeostasis. A defining characteristic of hepatic biology is its remarkable regenerative capacity following partial resection or chemical injury [1–4]. Hepatic regeneration is a complex, spatiotemporally orchestrated process in which the remodeling of the intrahepatic vascular network plays a critical role; restoration of angiogenesis and vascular perfusion represents a rate-limiting determinant of successful hepatic recovery [5–9]. Vascular dysfunction underlies the progression of clinically significant conditions including cirrhosis, acute liver failure, and small-for-size syndrome, underscoring the need for quantitative, longitudinal assessment of hepatic vascular dynamics during regeneration.

Noninvasive imaging has become indispensable for investigating hepatic regeneration in preclinical small animal models [10–12]. Micro-computed tomography (micro-CT) and MRI provide high spatial resolution but are limited by ionizing radiation exposure and prohibitive imaging costs, respectively, precluding high-frequency longitudinal monitoring [13,14]. Conventional ultrasound offers limited vascular contrast and frequently requires exogenous contrast agents [15–17]. Pure optical modalities such as fluorescence imaging are constrained by strong tissue scattering, limiting their utility for high-resolution imaging of deep hepatic structures [12,18]. Photoacoustic computed tomography (PACT) addresses these limitations by combining optical contrast with ultrasonic penetration depth, enabling label-free, high-resolution visualization of deep-tissue vasculature through endogenous hemoglobin absorption, with sensitivity to hemoglobin concentration, oxygen saturation, and hemodynamic changes [19–23].

In the present study, a custom 3-dimensional panoramic photoacoustic computed tomography (3D-PanoPACT) system incorporating a 1,024-element hemispherical transducer array and a 1,064-nm pulsed laser was employed to noninvasively monitor hepatic vascular remodeling throughout the compensatory remodeling trajectory following 70% partial hepatectomy (PHx) in a murine model [24,25]. The system provided whole-organ volumetric imaging with a 12-cm field of view without exogenous contrast agents, enabling longitudinal tracking of 3D vascular morphological parameters—including vascular volume, vessel length, mean diameter, and branching complexity—within the same animal across a 16-d regenerative period, thereby eliminating inter-individual variability inherent to cross-sectional sacrifice-based study designs. Functional hepatic metabolism was additionally assessed using the near-infrared II (NIR-II) contrast agent A1094, enabling quantitative evaluation of hepatobiliary clearance kinetics alongside structural vascular characterization. Histological validation with hematoxylin and eosin (H&E) staining and CD31 (platelet endothelial cell adhesion molecule-1 [PECAM-1]) immunohistochemistry was performed at day 16 to confirm tissue-level vascular restoration. Crucially, comprehensive serum biochemical analyses were performed to independently corroborate these functional findings and confirm systemic hepatobiliary compensation. This integrated imaging and quantification framework provides a comprehensive and dynamic characterization of the spatiotemporal progression of hepatic vascular remodeling, offering a rigorous experimental basis for understanding the vascular mechanisms underlying hepatic recovery.

## Results

### 3D-PanoPACT system characterization and baseline vascular assessment

Hepatic regeneration post-70% PHx progresses from a proliferative phase—driven by portal flow redistribution and shear-stress-induced angiogenesis—to a termination phase marked by vascular morphological stabilization (Fig. 1A). To noninvasively monitor this process, we developed a 3D-PanoPACT system featuring a 1,024-element hemispherical transducer array and a 1,064-nm pulsed laser (Fig. 1B and Video S1). Image quality was optimized via 1- to 6-MHz band-pass filtering and sinogram denoising (Fig. S1A to D), with system specificity validated by the absence of signal from nonabsorbing surgical sutures (Fig. S2A to C).

**Fig. 1.**
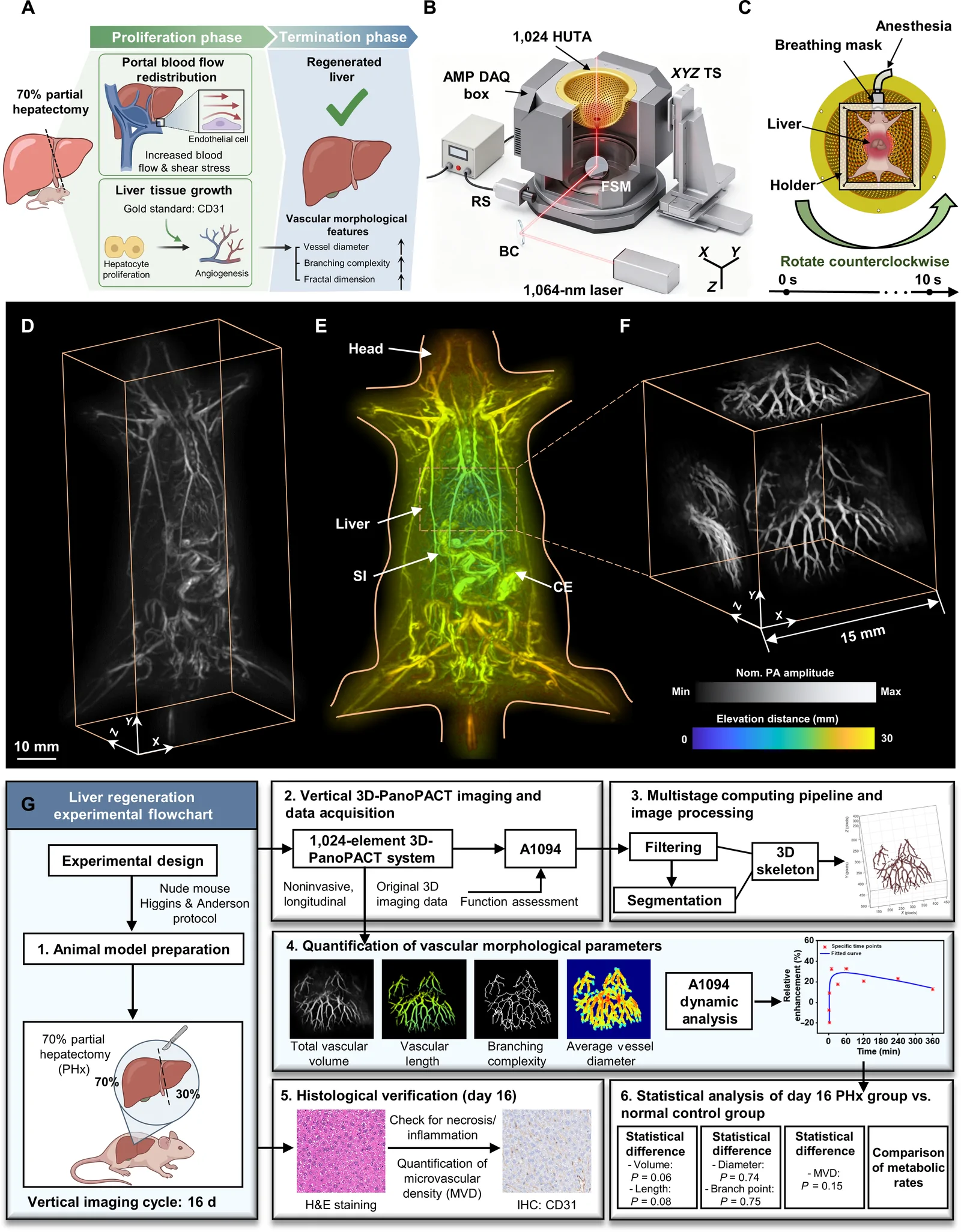
In vivo 3-dimensional panoramic photoacoustic computed tomography (3D-PanoPACT) framework and baseline characterization of the murine liver. (A) Schematic diagram of liver regeneration. (B) Schematic diagram of the 3D photoacoustic (PA) imaging setup. 1,024 HUTA, 1,024-element hemispherical ultrasonic transducer array; AMP, amplification circuits; DAQ, data acquisition module; RS, rotation stage; BC, beam combiner; FSM, front-silvered mirror; TS, translation stage. (C) Schematic diagram of mouse imaging and anesthesia and rotation method. (D) 3D PA image of the abdomen of a normal mouse. (E) Depth-encoded image of the entire abdomen of a mouse with a normal liver. SI, small intestine; CE, cecum. (F) PA images of the *XY*, *YZ*, and *XZ* fields of view of a normal mouse liver. (G) Flowchart of the liver regeneration experiment.

During imaging, prone-positioned nude (NU) mice underwent a 10-s rotation cycle to enhance volumetric reconstruction (Fig. 1C and Video S2). Respiratory artifacts were mitigated using a correlation-based gating strategy (Pearson coefficient > 0.85; Fig. S3). The system achieved high-contrast, label-free 3D visualization of systemic vasculature and abdominal organs (Fig. 1D to F and Video S3). Depth-encoded rendering localized hepatic vessels 10 to 20 mm from the ventral surface, distinctly delineating the small intestine and cecum (Fig. 1E). Magnified views (field of view: 15 mm) resolved intrahepatic trunks and ramifying branches (Fig. 1F). The integrated workflow spans animal preparation, image processing, vascular quantification, metabolic assessment, and histology (Fig. 1G).

### Morphological and vascular comparison between normal and regenerated livers

Following 70% PHx (Fig. 2A), day 16 whole-body 3D PACT revealed pronounced visceral reorganization (the abdomen was 0 mm, and the back was 30 mm): the regenerated right lobe displaced laterally, while intestines migrated anteriorly into the resection cavity. These in vivo macroscopic shifts were fully corroborated by ex vivo anatomical assessments (Fig. 2B and Fig. S4).

**Fig. 2.**
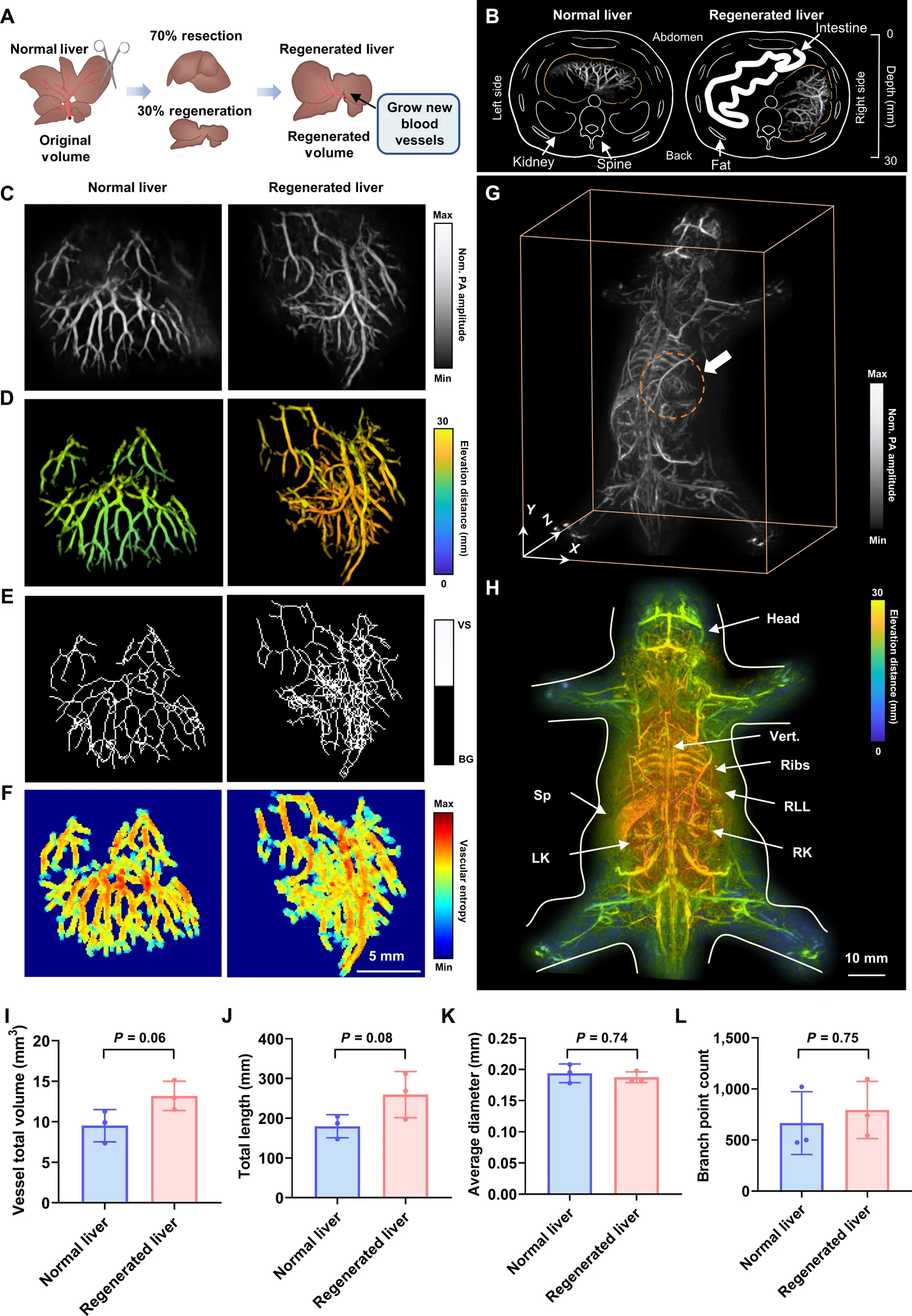
Comparison of normal and regenerated livers in mice. (A) Schematic diagram of a mouse liver regeneration model. (B) Comparison of the locations of normal and regenerated livers in mice. (C) Photoacoustic (PA) images, (D) depth-encoded images, (E) vascular skeleton (VS, vascular skeleton; BG, background), and (F) vascular entropy of normal and regenerated livers in mice. (G) Three-dimensional (3D) PA image of the back of a mouse with a regenerated liver. (H) Depth-encoded image of the dorsal region of a mouse with regenerated liver. Vert., vertebrae; Sp, spleen; RLL, right lobe of the liver; LK, left kidney; RK, right kidney. (I) Total vascular volume, (J) total vascular length, (K) average vascular diameter, and (L) vascular branching points of the normal and regenerated livers. Data are presented as mean ± SD (*n* = 3).

Photoacoustic (PA) images demonstrated enhanced structural complexity in the regenerated hepatic vasculature (Fig. 2C). Depth-encoded projections showed regenerated vessels expanding from deep hilar trunks toward the subcutaneous layer, diverging from the spatially confined distribution in normal livers (Fig. 2D). Skeletonization and entropy mapping highlighted distinct architectural shifts: normal livers presented 3 dominant trunks with systematic branching, whereas regenerated livers featured 2 primary trunks supplying a dense, fine vascular network (Fig. 2E and F). Dorsal imaging precisely localized the regenerated right lobe (15- to 25-mm depth, white arrow) and clearly delineated adjacent viscera (Fig. 2G and H and Video S4), showing a marked difference from the right side of the back of a normal mouse (Fig. S5 and Video S5), with a distinct area of regenerating liver vessels.

Quantitative 3D PA analysis at day 16 indicated compensatory vascular remodeling. No significant differences were observed between normal and regenerated livers in total vascular volume (*P* = 0.06), vessel length (*P* = 0.08), mean diameter (*P* = 0.74), or branching point density (*P* = 0.75) (Fig. 2I to L). Notably, mean vessel diameter remained consistent across normal and day 16 groups, reflecting a uniform, physiological growth pattern rather than aberrant hypertrophic remodeling.

### Longitudinal monitoring of hepatic vascular remodeling

Serial 3D PACT (days 2 to 16) captured 3 phases of vascular remodeling (Fig. 3A), with body weight monitoring confirming physiological recovery (Fig. S6).

**Fig. 3.**
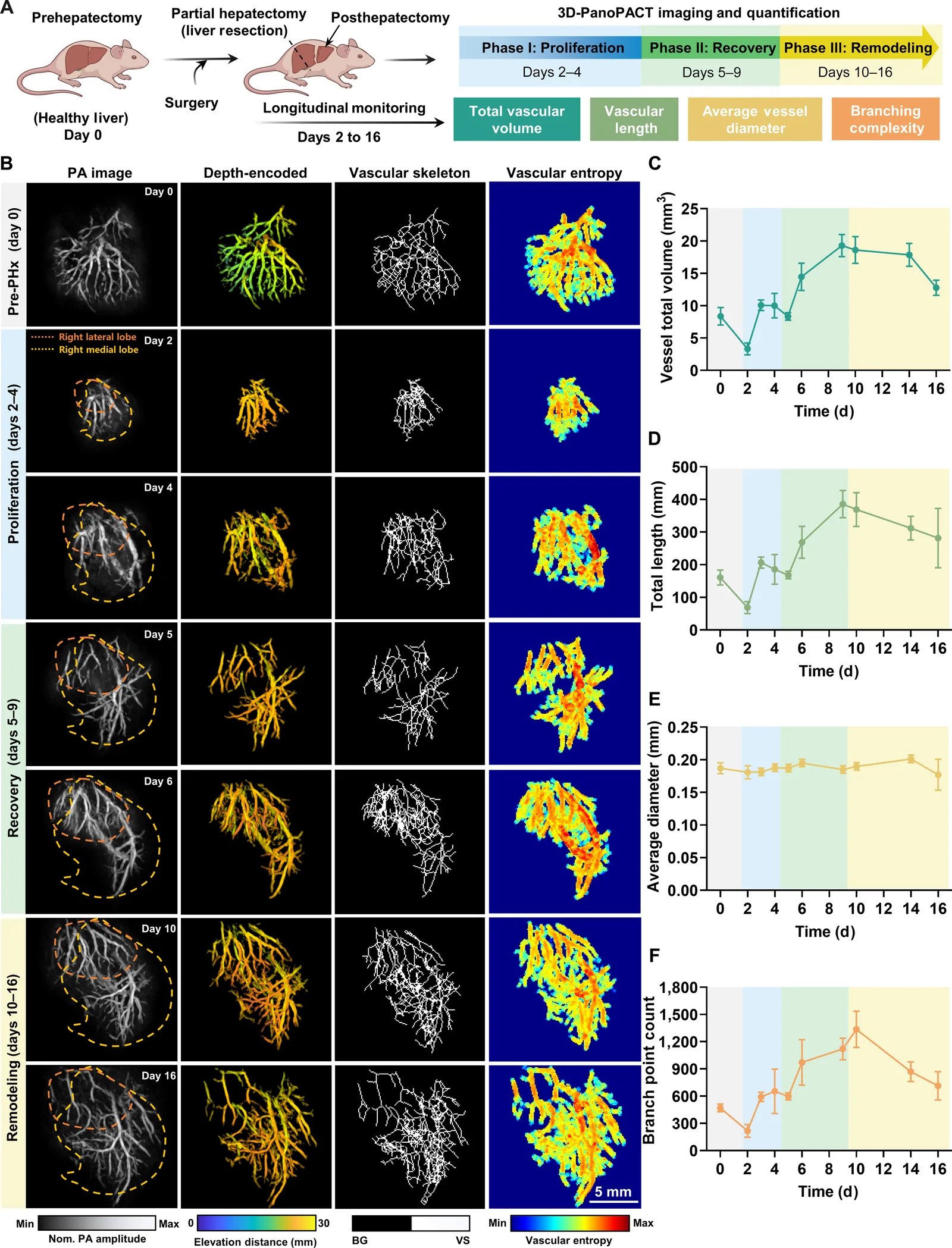
Longitudinal visualization of hepatic vascular remodeling after partial hepatectomy (PHx). (A) Experimental timeline and the retained phase framework: phase I, proliferation, days 2 to 4; phase II, recovery, days 5 to 9; and phase III, remodeling, days 10 to 16. Day 0 denotes imaging before resection in the longitudinal mice. (B) Representative photoacoustic (PA) images, depth-encoded images, vascular skeletons, and vascular entropy at the labeled times. (C) Total vascular volume, (D) total vascular length, (E) average vascular diameter, and (F) vascular branching points of the longitudinal mice. Data are presented as mean ± SD (*n* = 3 per group).

• Proliferative phase (days 2 to 4): Postresection volume loss was followed by progressive trunk thickening and sprouting by day 4. Entropy mapping identified a single dominant trunk driving initial perfusion (Fig. 3B).
• Recovery phase (days 5 to 9): Rapid expansion of the right lateral lobe featured a denser network. Depth-encoded imaging traced neovascularization extending from the hilum to the subcutaneous layer, coordinated by a second emergent trunk (Fig. 3B).
• Remodeling phase (days 10 to 16): Following inner lobe vascular expansion at day 10, structural pruning established a homeostatic configuration resembling normal vasculature by day 16 (Fig. 3B).

Quantitative curves revealed a surge in vascular volume during the proliferative phase (Fig. 3C), followed by increases in volume, diameter, and branching density during the recovery phase (Fig. 3C to F). Average vessel diameter remained stable throughout (Fig. 3E), reflecting a compensatory equilibrium between trunk maintenance and neovascular outgrowth.

To statistically substantiate the observed growth-to-remodeling pattern, we evaluated the phase means and within-phase slopes across the 3 regenerative phases (Fig. S7A to D and Tables S1 to S3). Phase I represents the early acute stage of regeneration, characterized by the initiation of rapid vascular growth with sharply increasing structural metrics. Following the active vascular expansion in phase II, the transition to phase III is characterized by a morphological shift toward structural network remodeling. Quantitatively, while phase III maintains a high overall mean (reflecting the expanded network established in phase II), its within-phase slope demonstrates a plateau or a slight decline. This complete statistical trajectory—an initial surge in phase I and phase II, followed by a high mean accompanied by a stabilizing slope in phase III, corroborates the morphological observation that the liver transitions from rapid anatomical growth into a functional remodeling phase.

### Validation of relative changes in longitudinal vascular remodeling in the sham-surgery group

To verify that the large-scale remodeling of the hepatic vascular network is a spontaneous regenerative process induced by liver lobe resection, rather than a nonspecific stress response caused by surgical trauma, this study introduced a sham-surgery group for longitudinal comparison within the same time period (Fig. S8). Mice in the sham group underwent the exact same procedures as those in the liver resection group—including anesthesia, laparotomy, liver exposure and manipulation, and laparoclosure—but no liver lobes were resected (Fig. S8A). We performed longitudinal photoacoustic imaging (PAI) tracking of the right hepatic region in mice in the sham group at time points matching those of the experimental group (Fig. S8B) and systematically quantified the total vascular volume, total vascular length, average vascular diameter, and number of vascular branching points (Fig. S8C to F). The quantitative data indicate that in the absence of liver resection, surgical trauma alone, organ manipulation, and repeated imaging procedures did not cause significant expansion of the hepatic vascular network morphology. The measurement data from the right liver in these sham-surgery groups, serving as an important local reference, strongly demonstrate that the intense vascular proliferation and structural changes observed in the liver resection model are spontaneous regenerative responses initiated by the liver to compensate for parenchymal loss, thereby ruling out interference from open surgery and other experimental interventions.

In addition, statistical results indicate that over the 16-d observation period, although there was some individual variability in the vascular parameters of the sham group, no significant overall time effect was observed (repeated-measures analysis of variance [ANOVA]; all adjusted *P* > 0.05) (Table S4). In contrast, by day 16, the relative changes (Table S5) in vascular volume and length in the liver resection group, compared to their preoperative baselines, reached +54.75% ± 23.45% and +78.14% ± 68.55%, respectively, demonstrating compensatory remodeling trajectory.

### Functional metabolic assessment using contrast-enhanced PACT

To evaluate functional recovery, hepatobiliary clearance kinetics were assessed using the NIR-II contrast agent A1094 (Fig. 4A). A1094 exhibits selective optical absorption at 1,064 nm, minimizing interference from endogenous chromophores (oxyhemoglobin, deoxyhemoglobin, and lipids) and ensuring contrast-specific PA detection (Fig. 4B).

**Fig. 4.**
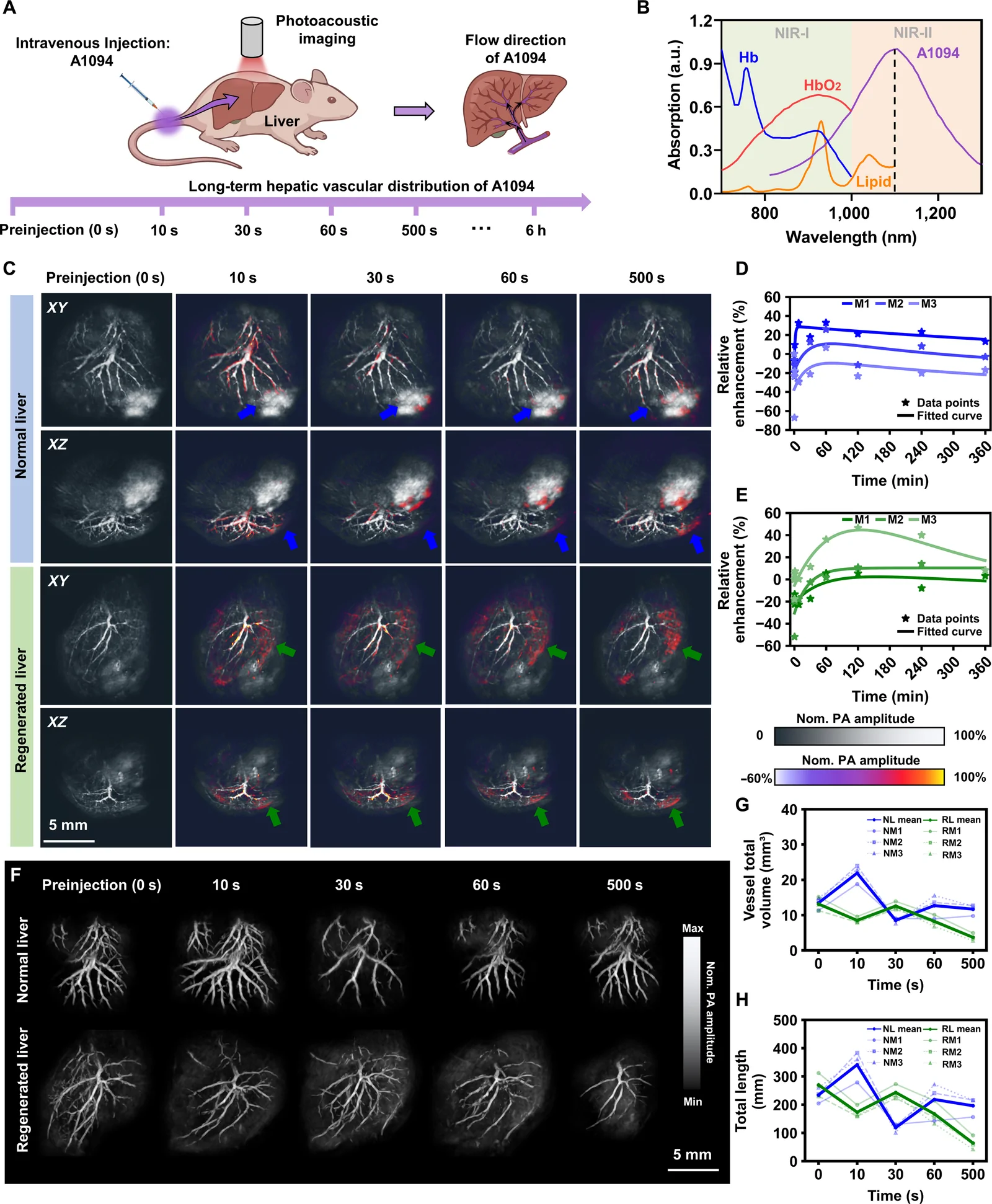
A1094-associated photoacoustic (PA) signal and vascular visualization dynamics in normal and regenerated livers. (A) Schematic of A1094 administration and the proposed hepatic transport process. (B) Optical absorption spectra; HbO_2_, oxyhemoglobin; Hb, deoxyhemoglobin. Imaging wavelength, 1,064 nm. (C) Representative *XY* and *XZ* views at the labeled pre- and postinjection times. Normal livers were imaged from the abdominal aspect; regenerated livers were imaged from the right dorsal aspect. (D and E) Individual liver-region relative-intensity observations and fitted curves over the 360-min observation period in normal and regenerated mice, respectively (*n* = 3 per group). (F) Representative regenerated-liver vascular images. (G and H) PA-visible vascular volume and total length, showing individual trajectories and group means (*n* = 3 per group).

Time-resolved PAI revealed characteristic spatiotemporal redistribution within 500 s postinjection: initial vascular enhancement transitioned to perisinusoidal parenchymal accumulation (blue arrow and green arrow), reflecting hepatocellular uptake (Fig. 4C). Extended monitoring (30 to 360 min) confirmed sustained accumulation followed by progressive signal attenuation, indicative of hepatobiliary excretion (Fig. S9). Quantitative metabolic curves demonstrated that relative PA intensity peaked between 30 and 60 min in both normal and regenerated livers before gradually declining (Fig. 4D and E).

Vascular kinetic analysis revealed peak intravascular enhancement at ~10 s in normal livers and ~30 s in regenerated livers (Fig. 4F), suggesting a moderately prolonged hepatic transit time postregeneration. During the initial perfusion phase, vascular volume and mean vessel diameter maximized as A1094 filled trunk vessels and microvasculature. Subsequent reductions in these parameters, alongside decreasing branching point density, corroborated the continuous parenchymal translocation and intravascular clearance of the contrast agent (Fig. 4G and H).

In the dynamic contrast-enhanced imaging, it should be noted that the nominal 10- and 30-s observation points represent the centers of 10-s volume acquisitions; thus, each image integrates the PA signal over this acquisition window. As shown in the supplementary group-level analysis (Fig. S10 and Table S6), the regenerated liver exhibited a later sampled vascular maximum compared to the normal state. Furthermore, extended continuous monitoring using A1094 over a 6-h period revealed a distinct rise followed by a lower fitted signal, a trajectory compatible with changing contrast distribution and gradual removal from the imaged region.

To independently evaluate hepatobiliary function, we performed dynamic contrast-enhanced PAI using indocyanine green (ICG) (Fig. S11A) [21,26]. The time–intensity curves revealed distinct initial perfusion dynamics: the normal liver exhibited a rapid signal maximum at approximately 10 s, whereas the regenerated liver showed a delayed time to peak at approximately 86 s (Fig. S11B and C). This temporal shift reflects the altered macroscopic contrast distribution and local hemodynamics within the remodeled vascular network. However, despite this difference in early perfusion, both the normal and regenerated livers successfully metabolized and cleared the ICG within a short period, with the PA signals fully returning to their respective preinjection baseline levels. These findings demonstrate that ICG clearance serves as an independent functional assessment, verifying that the regenerated liver has functionally compensated and restored its hepatobiliary excretory capacity.

### Histological and biochemical validation

Histological analysis was performed at day 16 to provide tissue-level validation of the PACT findings (Fig. 5A). Gross morphological comparison confirmed compensatory regrowth of the remnant lobe relative to normal controls (Fig. 5B and C). H&E staining revealed marked heterogeneity in vascular caliber within regenerated-liver sections (red arrow), in contrast to the uniform vessel diameters of normal-liver tissue, with high-magnification views demonstrating frequent hepatocyte binucleation and hepatic cord thickening indicative of active regenerative proliferation and no evidence of inflammation, necrosis, or hepatocellular injury (Fig. 5D). Immunohistochemistry (CD31) demonstrated comparable endothelial distribution patterns between groups (Fig. 5E), with no statistically significant difference in vascular density (*P* = 0.15; Fig. 5C), corroborating the PACT-derived finding of restored vascular parameters at day 16 and collectively confirming functionally and structurally compensatory remodeling trajectory following 70% PHx. The macroscopic compensatory growth of the right liver lobe (Fig. S4) and the recovery of total liver mass (Fig. S6) align perfectly with our 3D-PACT volumetric findings, confirming that internal vascular remodeling is the structural driver of gross anatomical recovery.

**Fig. 5.**
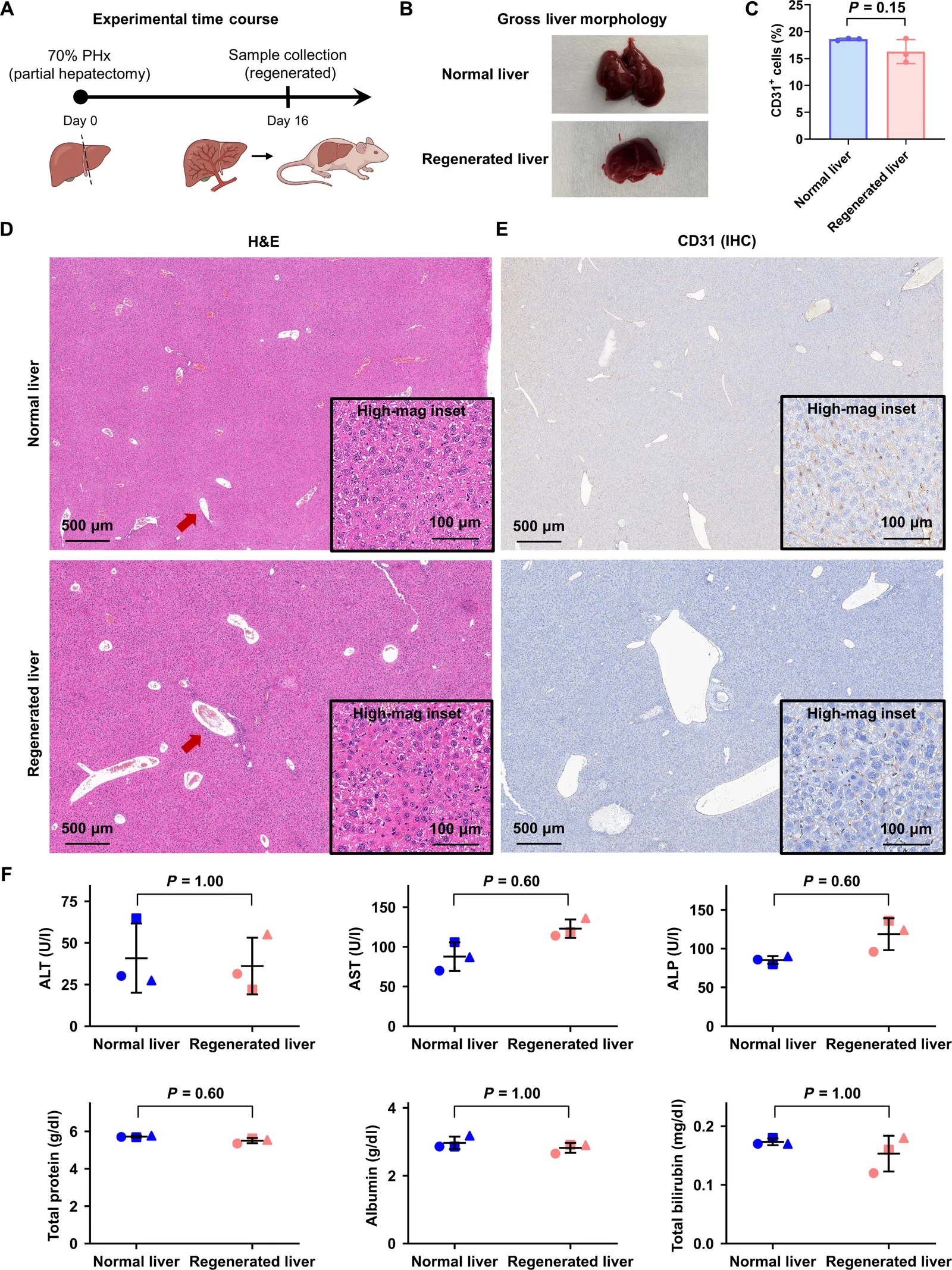
Histological and biochemical validation of regenerated and normal mouse livers. (A) Experimental time course of regenerated mouse livers. (B) Gross liver morphology of normal and regenerated livers. (C) Percentage of CD31-positive cells in sections. (D and E) Hematoxylin and eosin (H&E) and immunohistochemistry (CD31) results for normal and regenerated livers. (F) Comparison of serum alanine aminotransferase (ALT), aspartate aminotransferase (AST), alkaline phosphatase (ALP), total protein, albumin, and total bilirubin. Blue represents the control group, and pink represents the regenerative group. *P* values were calculated using 2-sided exact permutation tests and adjusted using the Holm method across the 6 biochemical endpoints. Data are presented as mean ± SD (*n* = 3).

To independently evaluate hepatobiliary functional recovery [27] following PHx, we assessed the comprehensive serum biochemical profiles (Fig. 5F) of normal mice and regenerated-liver mice at postoperative day 16 (*n* = 3 per group). The panel included markers of hepatic injury (alanine aminotransferase [ALT] and aspartate aminotransferase [AST]), biliary excretion (alkaline phosphatase [ALP] and total bilirubin [TBIL]), and synthetic capacity (total protein and albumin). While minor variations were observed—such as modestly lower synthetic markers and higher mean AST and ALP in the regeneration group—these remained within comparable physiological ranges (e.g., ALT: 36.17 ± 17.06 U l^−1^ vs. 40.83 ± 20.80 U l^−1^; TBIL: 0.153 ± 0.031 mg dl^−1^ vs. 0.173 ± 0.006 mg dl^−1^ for regenerated vs. normal mice, respectively). Importantly, 2-sided exact permutation tests with Holm correction confirmed no statistically significant differences between the 2 groups across all 6 quantifiable endpoints. These biochemical measurements provide robust, complementary evidence that the residual liver has achieved adequate hepatobiliary functional compensation by day 16, independently corroborating our in vivo PA observations.

## Discussion

The present study establishes 3D-PanoPACT as the first noninvasive, longitudinal, label-free modality for monitoring 3D hepatic vascular remodeling throughout the 70% PHx regenerative process. While recent CT and MRI advances improve anatomic assessments, they restrict high-frequency longitudinal monitoring [28–30]. Similarly, contrast-enhanced ultrasound imaging provides real-time data but lacks 3D vascular spatial resolution [5]. Overcoming these limitations, 3D-PanoPACT achieved ~0.25- to 0.30-mm isotropic resolution, enabling organ visualization of resolvable hepatic vessels and their branches without exogenous contrast. This technical superiority was validated by robust noise suppression and consistent anatomical fidelity (Fig. S6).

A central finding is the in vivo delineation of 3 distinct vascular remodeling phases: proliferative (days 2 to 4), recovery (days 5 to 9), and remodeling (days 10 to 16). This progression aligns with established hemodynamic-driven regenerative frameworks [20,31]. Crucially, our imaging revealed a hierarchical angiogenic strategy: the emergence of a primary dominant trunk followed by a second, supporting the concept of vascular guidance tunnels [32]. Depth-encoded mapping further captured a centrifugal neovascularization pattern extending from the hilum to the subcutaneous periphery. Our 3-stage framework successfully maps the complete trajectory of compensatory vascular remodeling in the liver. However, when interpreting the final state of this remodeling (stage 3), 2 objective limitations must be clarified: First, at the biological level, there is an inherent difference in anatomical coverage between the residual liver undergoing compensatory hyperplasia and the intact native liver on day 0. Second, at the statistical level, given the current sample size (*n* = 3), the lack of statistically significant differences in end-of-monitoring period indicators cannot be formally equated with achieving “equivalence” to the normal state. Based on these dual considerations, the longitudinal quantitative indicators in this study are strictly used to characterize the macrolevel dynamic trends of vascular remodeling, rather than to claim absolute and complete restoration of the liver’s anatomical structure.

Additionally, functional metabolic assessment using the NIR-II agent A1094 successfully tracked hepatobiliary clearance capacity. The delayed peak intravascular enhancement in regenerated livers (30 s vs. 10 s in normal livers) reflects an altered vascular architecture and prolonged transit time. The spatiotemporal transition from the intravascular compartment to parenchymal accumulation and subsequent hepatobiliary excretion (30 to 360 min; Fig. S9) confirmed intact hepatocellular transport [33,34]. The concomitant decline in quantitative vascular metrics during clearance validated these parameters as sensitive functional biomarkers [35,36]. While we observed a temporal shift in the vascular signal maximum (e.g., at 30 s in the regenerated liver versus 10 s in the normal liver), this difference should be interpreted with specific constraints. Since our segmented endpoints describe the PA-visible network rather than absolute plasma contrast concentration, this delayed maximum reflects the integrated contrast dynamics within the localized liver region. Therefore, this observed temporal difference delineates a shift in macroscopic contrast distribution, rather than denoting an exact 3-fold reduction in absolute blood flow or a directly quantified prolongation of physiological transit.

Critically, the PACT-derived stabilization of vascular parameters by day 16 was rigorously corroborated by macroscopic mass recovery (Figs. S4 and S6) and histology. H&E staining showed no necrotic injury, and immunohistochemistry (CD31) confirmed comparable vascular densities (*P* = 0.15), providing multiscale evidence of compensatory remodeling trajectory. Furthermore, the absence of significant differences in comprehensive serum biochemical profiles between the regenerated and normal mice independently confirms that the macroscopic vascular remodeling successfully translates into adequate hepatobiliary functional compensation.

Several limitations warrant consideration. A primary limitation of our study is the spatial resolution constraint (~0.2 to 0.3 mm) of in vivo deep-tissue imaging. Subresolution hepatic capillary networks generate a diffuse biological background that can introduce segmentation biases, such as overestimating vessel diameters, merging adjacent structures, or missing low-contrast microbranches. Importantly, this resolution threshold influences how we interpret longitudinal regeneration. The gradual increases in measured vascular volume and length likely reflect both macroscopic vessel growth and the densification of microvascular beds that collectively surpass the detection threshold. Consequently, our extracted metrics represent the morphological expansion of the resolvable macroscopic network, rather than individual capillary proliferation. Despite this limitation, the noninvasive PA framework remains highly robust for evaluating global hepatic vascular remodeling.

In addition, longitudinal and metabolic observations relied on representative animals; larger cohorts are needed for robust statistical profiling and quantitative pharmacokinetic modeling. While sufficient for murine models, the current imaging depth requires optimization for large-animal or clinical translation. Nonetheless, 3D-PanoPACT emerges as a uniquely capable platform for evaluating solid organ regeneration and preclinical proregenerative interventions.

## Materials and Methods

### Technical design of the PACT imaging system

The 3D-PanoPACT system incorporated a 1,024-element hemispherical ultrasonic transducer array (3.5C3.5-SR100-1024; ULSO TECH Co., Ltd., China) with a center frequency of 3.16 MHz and a bandwidth of 3.67 MHz. Acoustic excitation was driven by a 1,064-nm neodymium-doped yttrium aluminum garnet pulsed laser (SGR-S500; Beamtech Optronics Co., Ltd., Beijing, China) operating at 10 Hz. Uniform illumination was delivered via an engineered diffuser (EDC-50; RPC Photonics Inc., Rochester, NY, USA) across a 12-cm field of view. The maximum laser fluence on the skin surface was approximately 12.5 mJ cm^−2^, well within the American National Standards Institute safety limit of 100 mJ cm^−2^ for 1,064-nm wavelengths.

During imaging, NU mice were secured in a customized holder. Anesthesia was induced at 2% to 3% and maintained at 1.0% to 1.5% using isoflurane (RWD Life Science, Shenzhen, China) delivered via a nose cone. Acoustic coupling was maintained in a 25 °C water bath. Raw signals were digitized at a 40-MHz sampling rate using a data acquisition system (Legion ADC; PhotoSound Technologies, Houston, TX, USA) and subsequently processed in MATLAB R2025a (MathWorks, Natick, MA, USA) for volumetric reconstruction and quantitative analysis.

### Spatial resolution characterization

System spatial resolution was characterized using polystyrene microspheres as point targets (Fig. S12A). Following volumetric reconstruction, normalized intensity profiles were extracted along the *X*, *Y*, and *Z* axes through the centroid of each reconstructed microsphere, and spatial resolution was defined as the full width at half maximum (FWHM) of the resulting point-spread function curves. Measured resolutions of 0.266, 0.247, and 0.299 mm along the *X*, *Y*, and *Z* axes, respectively, confirmed near-isotropic volumetric imaging performance (Fig. S12B to D).

### Animals and study approval

All animal experiments were conducted in accordance with the Guide for the Care and Use of Laboratory Animals and approved by the Institutional Animal Care and Use Committee of Hangzhou Medical College (Approval No. SYXK2019-0011). Male NU mice (age, 6 to 8 weeks; weight, 18 to 28 g) underwent 70% PHx following the Higgins and Anderson protocol [37]. Under isoflurane anesthesia, the median and left lateral lobes, comprising approximately 70% of total liver mass, were ligated with 4-0 silk sutures and excised via midline laparotomy. The abdominal wall was closed in layers following confirmation of hemostasis, and the residual hepatic remnant underwent compensatory regeneration over a 16-d observation period.

The A1094 solution (Thermo Fisher Scientific, Waltham, MA, USA) was prepared by dissolving 0.3 mg of solid A1094 in 20 μl of dimethyl sulfoxide (Thermo Fisher Scientific, Waltham, MA, USA), followed by dilution in 7 ml of distilled water and sonication for 5 min to ensure complete dissolution. For in vivo imaging, mice were administered A1094 via tail vein injection at a dose of 1 μmol kg^−1^ body weight (approximate injection volume, 0.4 ml).

### Combined translational–rotational scanning

Whole-body imaging employed a 7 × 10 step-and-scan grid (step sizes: 6 mm *X* and 8 mm *Y*). At each position, 10-Hz 360° rotational data were filtered via correlation-based gating. Local subvolumes, reconstructed using CUDA-accelerated back projection, were integrated globally. Stitching artifacts and intensity discontinuities were mitigated using a 3D Gaussian weighting mask (FWHM = 30 mm) and weighted average fusion (Video S6).

### Image fusion

The final processed image ($V_{\text{fused}}$) was generated by linear weighted fusion of the vessel-enhanced image (V) and the normalized original PA image ($I_{\mathrm{norm}}$) (Fig. S13A) according to

$$V_{\text{fused}}=\alpha \cdot V+\left(1-\alpha \right)\cdot I_{\mathrm{norm}}$$ (1)

where the weighting coefficient *α* was empirically set to 0.3 to highlight vascular connectivity while preserving parenchymal background for spatial reference (Fig. S13B).

### Image processing and quantitative vascular analysis

MATLAB-based processing of 3D PA volumes entailed Gaussian smoothing, 99.2nd percentile thresholding, morphological denoising (<1,000 voxels removed), and 0.5× downsampling. Vessels were enhanced using a Frangi filter and secondary topological denoising (<2,000 voxels), followed by topology-preserving skeletonization. Vascular complexity was quantified via a composite anisotropy index (combining 3D entropy and structure tensor decomposition). At a 0.05-mm isotropic resolution, 4 metrics were extracted: vascular volume, vessel length, mean diameter, and branching density. The MATLAB code used for image reconstruction and quantitative vascular analysis is publicly available at https://github.com/Mengfan0917/3D-PanoPACT-Liver-Regeneration.git.

Although a computational voxel spacing of 0.05 mm was employed for 3D reconstruction and image processing, the intrinsic physical spatial resolution of our 3D-PanoPACT system is approximately 0.2 to 0.3 mm. Consequently, microvascular structures smaller than the system’s point-spread function cannot be individually distinguished. The extracted structural metrics—such as apparent vessel diameter, volume, length, and branching points—must therefore be strictly interpreted as macroscopic properties of the resolvable, PA-visible vascular network, rather than direct anatomical measurements of individual microscopic capillaries or hepatic sinusoids.

### Sham procedure and imaging

Under isoflurane (1.5% concentration) anesthesia, mice underwent laparotomy, exposure, and manipulation of the liver without removal of any lobe, followed by closure. Day 0 imaging was obtained before the procedure. Only the right liver was included in the sham imaging region. Serial quantitative measurements were obtained at days 0, 2, 3, 4, 5, 6, 9, 10, 14, and 16, with selected image time points shown in the figure.

### In vivo functional evaluation of liver using ICG clearance

A 1 mg ml^−1^ ICG solution was administered through the tail vein at 5 mg kg^−1^, followed by pre- and postinjection PAI at 750 nm. The normal liver was imaged from the abdominal aspect and the regenerated liver from the right dorsal aspect. The final figure contains one normal-liver animal and one regenerated-liver animal.

### Histological analysis

Following completion of the 16-d imaging period, liver tissues from both PHx and control mice were harvested, fixed in 4% paraformaldehyde, and paraffin-embedded for sectioning at 4-μm thickness. H&E staining was performed to evaluate general morphological changes, and immunohistochemical staining for CD31 was performed to assess vascular density and endothelial distribution. Histological slides were digitized using a high-resolution digital microscope for correlation with the 3D PACT findings.

### Serum biochemical analysis

To further evaluate hepatobiliary function and assess potential liver injury, serum biochemical analyses were conducted on normal mice (*n* = 3) and regenerated mice (*n* = 3, at day 16 postresection). Blood samples were collected via retro-orbital bleeding under general anesthesia. The collected whole blood was allowed to clot at room temperature for 30 min, followed by centrifugation at 3,000 *g* for 15 min at 4 °C to isolate the serum. The serum samples were then analyzed using an automated biochemical analyzer (AU5800, Beckman Coulter, USA). The quantitative panel included ALT, AST, ALP, gamma-glutamyl transferase, total protein, albumin, and TBIL.

### Statistical analysis

Statistical analyses were performed using GraphPad Prism 9.0 (GraphPad Software, USA), with data presented as mean ± SD. For general cross-sectional comparisons, statistical significance (*P* < 0.05) was determined using the unpaired 2-tailed Student *t* test for 2 groups or one-way ANOVA followed by Tukey’s post hoc test for 3 or more groups. To evaluate the longitudinal dynamics of hepatic vascular remodeling, the observation period was delineated into 3 regenerative phases: phase I (days 2, 3, and 4), phase II (days 5, 6, and 9), and phase III (days 10, 14, and 16). For each animal, the arithmetic means and least-squares slopes of the quantitative metrics were calculated to characterize phase-specific parameter levels and dynamic trends. Pairwise interphase evaluations were subsequently performed using paired *t* tests. To control for multiple testing, Holm adjustment was applied across the 12 contrasts separately for means and slopes, alongside unadjusted pointwise 95% confidence intervals to illustrate estimate variability. Importantly, to address the intrinsic statistical constraints of the small sample size (*n* = 3), exact sign-flip tests enumerating all 8 possible sign combinations were incorporated. Given that the smallest possible 2-sided *P* value for 3 paired animals is 0.25, parametric and exact findings were evaluated concurrently. Consequently, the parametric results for these longitudinal phases were strictly interpreted as exploratory analyses.

## Data Availability

The data supporting this study’s findings are available from the corresponding authors upon reasonable request.

## Supplementary Materials

Supplementary 1 Figs. S1 to S13 Tables S1 to S6 Videos S1 to S6
