# Supplementary material for: Photoacoustic Tomography Tracks Vascular Remodeling and Functional Recovery in Liver Regeneration
Source: BME Front. 2026 Oct 21;7:0327. doi: 10.34133/bmef.0327 (PMC13683954; doi:10.34133/bmef.0327)
Supplement: Supplementary file 1 [file bmef.0327.f1.zip › Revised Supplementary Materials.docx]

**Supplementary Materials for**

Photoacoustic Tomography Tracks Vascular Remodeling and Functional Recovery in Liver Regeneration

*Corresponding author. Email: [xh-wang@zhejianglab.org](mailto:xh-wang@zhejianglab.org), junhuishi@outlook.com

The file includes:

Supplementary Figures S1 to S13

Supplementary Tables S1 to S6

Supplementary Movies S1 to S6

**Fig. S1.** Preprocessing and denoising of raw photoacoustic (PA) signals. (A) Power spectrum analysis (FFT) showing original (gray) and band-pass filtered (red, 1–6 MHz) signals, demonstrating effective suppression of low-frequency drift and high-frequency noise. (B) Representative time-domain signals (Channel 512) before (gray) and after (blue) filtering, indicating a significant improvement in the signal-to-noise ratio (SNR). (C) Raw sinogram (B-scan) illustrating strip artifacts and background noise. (D) Denoised sinogram with enhanced signal contrast and suppressed background, providing high-quality data for subsequent volumetric reconstruction.

**Fig. S2.** Performance verification using a non-photoacoustic phantom. (A) Schematic of the phantom: A surgical suture (Line) used for mouse ligation was embedded in an agar cylinder. Notably, this ligation line does not possess photoacoustic properties (i.e., no optical absorption at the excitation wavelength). (B) Raw data (Sinogram): The data captured by the 1024-element array. Only background noise and system interference are visible, with no characteristic photoacoustic signals (hyperbolic arcs) detected from the ligation line. (C) MIPs: Reconstructed images in XY, XZ, and YZ planes. The absence of visible structures confirms that the ligation line remains "invisible" in the photoacoustic modality, demonstrating the system's specificity and low background artifacts.

**Fig. S3.** Selection of static frames based on inter-frame correlation analysis. The correlation-based gating method was employed to identify frames with minimal motion artifacts. (Blue line) The normalized average correlation curve, where each point represents the mean Pearson correlation coefficient between a specific frame and all other frames within the acquisition sequence. (Red asterisks) Identified "static frames" that satisfy the pre-defined threshold (Correlation > 0.85). These frames exhibit high structural similarity and were selected for subsequent signal averaging and image reconstruction to suppress motion-induced blurring.

**Fig. S4.** In situ anatomical localization of normal versus regenerated livers.

**Fig. S5.** Whole-body PA imaging of a normal mouse. Yellow circle and white arrow indicate the anatomical location of the right liver lobe.

**Fig. S6.** Longitudinal changes in mouse body weight before and during liver regeneration. Body weights were recorded at Day 0 (pre-partial hepatectomy) and at indicated time points post-surgery to monitor the overall physiological recovery of the mice.

**Fig. S7.** Individual-animal phase summaries within the retained three-phase framework. (A) Total vascular volume, (B) total vascular length, (C) average vascular diameter, and (D) vascular branching points of the longitudinal mice. Data are presented as mean ± SD (n = 3 per group). Coloured points are the mean of the three observed values in each phase for one mouse; lines connect the same mouse. Black points and error bars represent mean ± SD across the three mice. The curves summarize phase levels, not within-phase slopes or statistically re-estimated transition times.

**Fig. S8.** Right-liver PA imaging after sham surgery. (A) Sham procedure comprising anesthesia, laparotomy, liver exposure/manipulation and closure without resection. Only the right liver was imaged; the panel does not represent an abdominal whole-liver measurement. (B) Representative right-liver images at the indicated preoperative and postoperative days. (C) Total vascular volume, (D) total vascular length, (E) average vascular diameter, and (F) vascular branching points of the longitudinal mice. Data are presented as mean ± SD (n = 3 per group). The sham right-liver measurements serve as a regional reference for the early residual right-liver measurements after resection, rather than requiring equality with normal abdominal whole-liver values.

**Fig. S9.** Enhanced images of liver regions showing metabolism over time (30 min, 60 min, 120 min, 240 min, 360 min) in normal and regenerated livers following administration of A1094 (XY and XZ views).

**Fig. S10.** Early baseline-relative vascular dynamics in normal and regenerated livers. Faint lines indicate individual animals; prominent curves and error bars indicate mean ± SD across three mice per group. Relative changes in segmented vascular volume and length are plotted against actual post-injection time. These are measurements of the PA-visible vascular network, not calibrated contrast concentrations.

**Fig. S11.** ICG-associated PA dynamics in normal and regenerated liver regions. (A) Representative XY views before injection and at the labelled post-injection times. Normal-liver images were acquired from the abdominal aspect; regenerated-liver images were acquired from the right dorsal aspect. Scale bars, 5 mm. (B,C) Relative-intensity observations (grey points) and the final fitted curves for the normal and regenerated animals, respectively. Blue and green denote normal and regenerated liver. ICG was administered at 5 mg/kg using a 1 mg/mL solution; imaging wavelength, 750 nm.

**Fig. S12.** Characterization of the system’s 3D spatial resolution. (A) Orthogonal MIPs (MIP: maximum intensity projection) of a point source in the XY, XZ, and YZ planes. Orange dashed lines indicate the cross-sectional positions used for profile analysis. Scale bar: 100 μm. (B–D) Normalized intensity profiles along the X, Y, and Z axes, respectively. The spatial resolution, quantified by the FWHM, was determined to be 0.266 mm (X-axis), 0.247 mm (Y-axis), and 0.299 mm (Z-axis).

**Fig. S13.** Comparison of raw and filtered PA images of a normal mouse liver. (A) Representative raw PA image. (B) PA image after denoising and filtering, showing suppressed background interference.

**Supplementary Tables：**

**Table S1.** Overall phase effects on vascular parameter means and within-phase slopes.

| **F statistic** | **Greenhouse–Geisser ε** | **GG-adjusted numerator df** | **GG-adjusted denominator df** | **GG-adjusted P value** | **Vascular parameter** | **Summary measure** | **Holm-adjusted P value** |
| --- | --- | --- | --- | --- | --- | --- | --- |
| 115.6884035 | 0.500669643 | 1.001339287 | 2.002678574 | 0.008493936 | Vessel Total Volume (mm^3^) | mean | 0.033975742 |
| 14.36346233 | 0.550696731 | 1.101393462 | 2.202786924 | 0.054223241 | Vessel Total Volume (mm^3^) | Slope per day | 0.216892964 |
| 25.42584758 | 0.500324965 | 1.000649931 | 2.001299862 | 0.037103495 | Total Length (mm) | mean | 0.111310485 |
| 7.420228284 | 0.516556697 | 1.033113393 | 2.066226786 | 0.108950304 | Total Length (mm) | Slope per day | 0.326850911 |
| 0.805819128 | 0.58898915 | 1.1779783 | 2.3559566 | 0.474299213 | Total Length (mm) | mean | 0.474299213 |
| 1.642608699 | 0.586045731 | 1.172091463 | 2.344182925 | 0.323897562 | Total Length (mm) | Slope per day | 0.326850911 |
| 21.16507444 | 0.509526462 | 1.019052925 | 2.03810585 | 0.042617796 | Branch Points Count | mean | 0.111310485 |
| 6.140447309 | 0.546412756 | 1.092825511 | 2.185651022 | 0.121863899 | Branch Points Count | Slope per day | 0.326850911 |

Overall phase effects were assessed using repeated-measures ANOVA with Greenhouse–Geisser (GG) correction (n = 3 mice). Numerator and denominator degrees of freedom are GG-adjusted. Holm adjustment was applied across the four vascular parameters separately for phase means and within-phase slopes.

**Table S2.** Pairwise comparisons of vascular parameter means and slopes between regenerative phases.

| **Vascular parameter** | **Summary measure** | **Phase comparison** | **Mean paired difference** | **Lower 95% CI** | **Upper 95% CI** | **Paired t-test P value** | **Exact sign-flip P value** | **Holm-adjusted paired t-test P value** | **Holm-adjusted sign-flip P value** |
| --- | --- | --- | --- | --- | --- | --- | --- | --- | --- |
| Vessel Total Volume (mm^3^) | mean | II minus I | 6.235659 | 6.139216 | 6.332102 | 1.29E-05 | 0.25 | 0.000155 | 1 |
| Vessel Total Volume (mm^3^) | mean | III minus II | 2.382291 | -0.68607 | 5.450649 | 0.07912 | 0.25 | 0.474719 | 1 |
| Vessel Total Volume (mm^3^) | mean | III minus I | 8.61795 | 5.521003 | 11.7149 | 0.006904 | 0.25 | 0.062132 | 1 |
| Vessel Total Volume (mm^3^) | Slope per day | II minus I | -0.86933 | -5.58007 | 3.841411 | 0.510431 | 0.75 | 1 | 1 |
| Vessel Total Volume (mm^3^) | Slope per day | III minus II | -3.33859 | -4.87181 | -1.80536 | 0.011201 | 0.25 | 0.123214 | 1 |
| Vessel Total Volume (mm^3^) | Slope per day | III minus I | -4.20792 | -7.89845 | -0.51739 | 0.039128 | 0.25 | 0.352148 | 1 |
| Total Length (mm) | mean | II minus I | 120.416 | 116.5392 | 124.2927 | 5.6E-05 | 0.25 | 0.000616 | 1 |
| Total Length (mm) | mean | III minus II | 46.88298 | -79.2155 | 172.9814 | 0.250791 | 0.25 | 1 | 1 |
| Total Length (mm) | mean | III minus I | 167.2989 | 38.37578 | 296.2221 | 0.030612 | 0.25 | 0.244899 | 1 |
| Total Length (mm) | Slope per day | II minus I | -7.65327 | -121.077 | 105.7703 | 0.798905 | 1 | 1 | 1 |
| Total Length (mm) | Slope per day | III minus II | -65.3738 | -83.9406 | -46.807 | 0.004329 | 0.25 | 0.051946 | 1 |
| Total Length (mm) | Slope per day | III minus I | -73.0271 | -177.451 | 31.39683 | 0.094975 | 0.25 | 0.676916 | 1 |
| Average Diameter (mm) | mean | II minus I | 0.005615 | -0.00616 | 0.017394 | 0.17673 | 0.25 | 0.883648 | 1 |
| Average Diameter (mm) | mean | III minus II | 0.000332 | -0.02246 | 0.023123 | 0.95577 | 1 | 1 | 1 |
| Average Diameter (mm) | mean | III minus I | 0.005947 | -0.02374 | 0.035628 | 0.479533 | 0.5 | 1 | 1 |
| Average Diameter (mm) | Slope per day | II minus I | -0.0047 | -0.01987 | 0.010482 | 0.314625 | 0.5 | 1 | 1 |
| Average Diameter (mm) | Slope per day | III minus II | -0.0002 | -0.00555 | 0.005149 | 0.885783 | 1 | 1 | 1 |
| Average Diameter (mm) | Slope per day | III minus I | -0.0049 | -0.02103 | 0.011234 | 0.321499 | 0.5 | 1 | 1 |
| Branch Points Count | mean | II minus I | 409.8889 | 358.5679 | 461.2099 | 0.000846 | 0.25 | 0.008457 | 1 |
| Branch Points Count | mean | III minus II | 76.11111 | -355.324 | 507.5463 | 0.527085 | 0.75 | 1 | 1 |
| Branch Points Count | mean | III minus I | 486 | 73.87181 | 898.1282 | 0.036718 | 0.25 | 0.257023 | 1 |
| Branch Points Count | Slope per day | II minus I | -106.744 | -636.613 | 423.1256 | 0.477436 | 0.75 | 1 | 1 |
| Branch Points Count | Slope per day | III minus II | -217.292 | -373.879 | -60.7052 | 0.026923 | 0.25 | 0.269234 | 1 |
| Branch Points Count | Slope per day | III minus I | -324.036 | -757.607 | 109.5358 | 0.084615 | 0.25 | 0.676916 | 1 |

Values represent within-animal paired differences between phases (n = 3 mice). For “Phase II − Phase I”, positive values indicate higher measurements in Phase II. Confidence intervals are pointwise, unadjusted 95% intervals from paired t-tests. Holm adjustment was applied across 12 comparisons separately for phase means and within-phase slopes. Exact sign-flip tests enumerate all eight sign combinations.

**Table S3.** Within-phase slopes and directions of vascular parameter changes across individual animals.

| **Vascular parameter** | **Regenerative phase** | **Mean slope** | **SD of slopes** | **Minimum slope** | **Maximum slope** | **Mice with positive slopes (n)** | **Mice with negative slopes (n)** |
| --- | --- | --- | --- | --- | --- | --- | --- |
| Vessel Total Volume (mm^3^) | I | 3.347588 | 1.355328 | 2.152097 | 4.819985 | 3 | 0 |
| Vessel Total Volume (mm^3^) | II | 2.478261 | 0.556082 | 1.956657 | 3.063369 | 3 | 0 |
| Vessel Total Volume (mm^3^) | III | -0.86033 | 0.305591 | -1.08398 | -0.51213 | 0 | 3 |
| Total Length (mm) | I | 58.51914 | 31.37143 | 33.06989 | 93.56905 | 3 | 0 |
| Total Length (mm) | II | 50.86587 | 14.62054 | 36.16473 | 65.40445 | 3 | 0 |
| Total Length (mm) | III | -14.508 | 10.94184 | -27.1423 | -8.12218 | 0 | 3 |
| Average Diameter (mm) | I | 0.003498 | 0.005381 | -0.00043 | 0.009631 | 2 | 1 |
| Average Diameter (mm) | II | -0.0012 | 0.001143 | -0.00194 | 0.000119 | 1 | 2 |
| Average Diameter (mm) | III | -0.0014 | 0.003086 | -0.00457 | 0.001595 | 1 | 2 |
| Branch Points Count | I | 218.5 | 157.4857 | 89.5 | 394 | 3 | 0 |
| Branch Points Count | II | 111.7564 | 58.35951 | 56.57692 | 172.8462 | 3 | 0 |
| Branch Points Count | III | -105.536 | 32.25067 | -130.571 | -69.1429 | 0 | 3 |

Within-phase slopes were estimated separately for each mouse by linear regression against the actual postoperative days. Values summarize slopes across three mice. Positive and negative slopes indicate increasing and decreasing fitted trends, respectively; they do not indicate statistical significance. Slope units are mm³/day for vascular volume, mm/day for vessel length and mean diameter, and counts/day for branch point count.

**Table S4.** Vascular parameters in the right liver of mice that underwent sham surgery were significantly affected by the overall time factor.

| **Metrics** | **F-value** | **GG-corrected P-value** | **Holm-corrected P-value** |  |
| --- | --- | --- | --- | --- |
| Total vascular volume (mm^3^) | 1.1915 | 0.3927 | 1.000 | |
| Total vascular length (mm) | 0.6054 | 0.5486 | 1.000 | |
| Average vascular diameter (mm) | 2.0541 | 0.2744 | 1.000 | |
| Branching points count | 0.5074 | 0.6356 | 1.000 | |

**Table S5.** Relative changes in hepatic vascular parameters at day 16 compared with preoperative measurements in the same mice.

| **Metrics** | **Relative change mean** | **Relative change SD** |  |
| --- | --- | --- | --- |
| Total vascular volume (mm^3^) | 54.749747 | 23.453076 | |
| Total vascular length (mm) | 78.135249 | 68.554083 | |

**Table S6.** Group-level summary and statistical comparisons of vascular visualization dynamics.

| **Vascular parameter** | **Dynamic endpoint** | **Normal mean** | **normal_SD** | **regenerated_mean** | **regenerated_SD** | **Mean difference (regenerated − normal)** | **Two-sided exact permutation P value** | **Holm-adjusted P value** |
| --- | --- | --- | --- | --- | --- | --- | --- | --- |
| Total Length (mm) | All points max (s) | 10 | 0 | 0 | 0 | -10 | 0.1 | 1 |
| Total Length (mm) | Change 10s pct | 45.58821 | 14.00075 | -35.5903 | 4.360761 | -81.1785 | 0.1 | 1 |
| Total Length (mm) | Change 30 minus 10 pp | -93.8674 | 18.83216 | 26.10928 | 2.157797 | 119.9767 | 0.1 | 1 |
| Total Length (mm) | Change 30s pct | -48.2792 | 12.4446 | -9.48102 | 5.429526 | 38.79818 | 0.1 | 1 |
| Total Length (mm) | Change 500s pct | -16.5475 | 7.424609 | -76.4725 | 7.004555 | -59.925 | 0.1 | 1 |
| Total Length (mm) | Change 60s pct | -8.14442 | 19.29197 | -38.1349 | 10.66971 | -29.9905 | 0.1 | 1 |
| Total Length (mm) | Post observed peak (s) | 10 | 0 | 30 | 0 | 20 | 0.1 | 1 |
| Total Length (mm) | Post peak change pct | 45.58821 | 14.00075 | -9.48102 | 5.429526 | -55.0692 | 0.1 | 1 |
| Total Length (mm) | Raw AUC 0-500 | 103711.4 | 23948.16 | 63466.64 | 16396.96 | -40244.8 | 0.2 | 1 |
| Total Length (mm) | raw AUC 0-60 | 12534.03 | 1689.185 | 12540.85 | 1772.696 | 6.822077 | 1 | 1 |
| Total Length (mm) | Relative net AUC 0-500 pct (s) | -6077.54 | 5809.127 | -26556.5 | 4132.173 | -20479 | 0.1 | 1 |
| Total Length (mm) | Relative net AUC 0-60 pct (s) | -645.323 | 359.124 | -1342.9 | 317.433 | -697.58 | 0.1 | 1 |
| Vessel Total Volume (mm^3^) | All points max (s) | 10 | 0 | 10 | 17.32051 | 0 | 1 | 1 |
| Vessel Total Volume (mm^3^) | Change 10s pct | 63.32802 | 7.959727 | -34.9028 | 4.841614 | -98.2308 | 0.1 | 1 |
| Vessel Total Volume (mm^3^) | Change 30 minus 10 pp | -98.6599 | 11.03676 | 31.09186 | 2.192871 | 129.7517 | 0.1 | 1 |
| Vessel Total Volume (mm^3^) | Change 30s pct | -35.3319 | 14.68184 | -3.81094 | 5.945171 | 31.52092 | 0.1 | 1 |
| Vessel Total Volume (mm^3^) | Change 500s pct | -12.9846 | 2.724241 | -71.9009 | 6.173541 | -58.9163 | 0.1 | 1 |
| Vessel Total Volume (mm^3^) | Change 60s pct | -6.75974 | 13.0317 | -37.1905 | 9.15484 | -30.4308 | 0.1 | 1 |
| Vessel Total Volume (mm^3^) | Post observed peak (s) | 10 | 0 | 30 | 0 | 20 | 0.1 | 1 |
| Vessel Total Volume (mm^3^) | Post peak change pct | 63.32802 | 7.959727 | -3.81094 | 5.945171 | -67.139 | 0.1 | 1 |
| Vessel Total Volume (mm^3^) | Raw AUC 0-500 | 6165.323 | 1179.996 | 3262.362 | 686.2704 | -2902.96 | 0.1 | 1 |
| Vessel Total Volume (mm^3^) | Raw AUC 0-60 | 799.0821 | 86.51998 | 631.5227 | 75.14127 | -167.559 | 0.2 | 1 |
| Vessel Total Volume (mm^3^) | Relative net AUC 0-500 pct (s) | -4378.54 | 2553.333 | -25176.8 | 3585.502 | -20798.3 | 0.1 | 1 |
| Vessel Total Volume (mm^3^) | Relative net AUC 0-60 pct (s) | -34.7725 | 269.8604 | -1176.67 | 319.2047 | -1141.9 | 0.1 | 1 |

Data are presented as mean ± SD for three independent mice per group. Dynamic endpoints were calculated separately for each mouse before group-level summarization. Mean differences were calculated as regenerated minus normal. Two-sided exact permutation P values were obtained by enumerating all 20 possible group-label allocations. Holm adjustment was applied across all 24 endpoint comparisons reported in this table.

Videos：

Video 1. The 1024-channel distribution of the 3D-PanoPACT hemispherical sensor.

Video 2. The 1024-channel hemispherical rotation of 3D-PanoPACT.

Video 3. A 3D PA image of the entire body, taken via abdominal scanning of a normal mouse.

Video 4. A 3D PA image of the entire body, taken from a scan of the back of a mouse with regenerated liver.

Video 5. A 3D PA photoacoustic image of a normal mouse's back, capturing the entire body.

Video 6. 3D-PanoPACT scans the mouse's entire body in a translational motion.
